# Digital therapeutics, for a few dollars less

**DOI:** 10.1192/j.eurpsy.2026.10317

**Published:** 2026-07-31

**Authors:** B. Bolea Alamanac, Toronto

**Affiliations:** Psychiatry, University of Toronto, Toronto, Canada

## Abstract

**Abstract:**

In the post-pandemic context, demand for mental health care has increased substantially worldwide, exceeding the capacity of traditional service delivery models. While integrated care and task-shifting approaches have partially addressed this gap, scalable solutions that reduce reliance on highly specialized staff are increasingly needed. Digital health technologies offer significant potential to improve access, efficiency, and equity in mental health care delivery.

Tools such as mobile apps, web-based platforms, wearable devices, and digital phenotyping can support diagnosis, monitoring, and intervention, particularly in settings where mental health professionals are scarce.

National health systems are obligated to deliver evidence-based care equitably and efficiently. However, evaluating and implementing digital mental health interventions presents unique challenges. Unlike pharmacological or psychological treatments, apps lack clear placebo equivalents, face high attrition rates, and often exclude individuals without access to compatible devices or sufficient digital literacy. As a result, evidence derived from traditional randomized controlled trials may not fully capture real-world effectiveness, usability, or reach, particularly among vulnerable and marginalized populations.

A key advantage of digital interventions is their capacity for continuous data collection at minimal cost. This enables ongoing evaluation before, during, and after implementation, analogous to post-marketing surveillance for medications but without reporting delays. Continuous monitoring of clinical outcomes, engagement, and potential harms—including privacy breaches, misuse, and unintended increases in healthcare utilization—should be a prerequisite for apps adopted by national health systems.

Robust evaluation frameworks are required to assess not only clinical outcomes and population reach, but also usability, acceptability, safety, and ethical use of data. Clear distinctions are needed between apps that claim therapeutic benefit and those supporting lifestyle or wellness goals, with corresponding regulatory standards. As app prescription becomes more common, dedicated expertise may be required to guide safe and effective use, similar to the role of pharmacists in medication management. Looking ahead, advances in artificial intelligence may further expand the role of digital tools in mental health care, reinforcing the need for adaptive, continuous, and system-level evaluation approaches.

**Disclosure of Interest:**

None Declared

